# Brief introduction in phenotypic and genetic differences of C57BL/6 and BALB/c mice substrains

**DOI:** 10.1002/ame2.70067

**Published:** 2025-09-03

**Authors:** Lan Zhao, Jie Wei, Bingfei Yue

**Affiliations:** ^1^ Division of Laboratory Animal Monitoring National Institutes for Food and Drug Control Beijing China; ^2^ China National Rodent Laboratory Animal Resources Center Beijing China

**Keywords:** BALB/c mice, C57BL/6 mice, genetic differences, phenotypic differences, substrains

## Abstract

Experimental mice play a critical role in biomedical research. The phenotype and application of different substrains vary due to genetic differentiation and variation. To ensure validity and reliability of results, it is imperative to adhere to standardized experiments and controls. This paper objectively reviews the origin, differentiation, and phenotypic and genetic differences between the C57BL/6 and BALB/c mouse substrains. Furthermore, an optimal selection strategy is proposed based on the genetic quality control technology to facilitate the precise application of these two mouse substrains.

## Graphical abstract

This article briefly reviews the origin, differentiation, and phenotypic and genetic differences between the C57BL/6 and BALB/c mouse substrains.

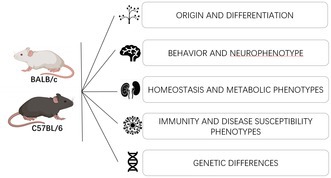

## 1 INTRODUCTION

Experimental animals serve as indispensable tools in biomedical research, enabling investigations into gene function, disease pathogenesis, and drug efficacy prior to clinical trials. Among these, mice are the most widely used due to their high genetic similarity to humans, low maintenance costs, and rapid reproduction rates.

**FIGURE 1:**
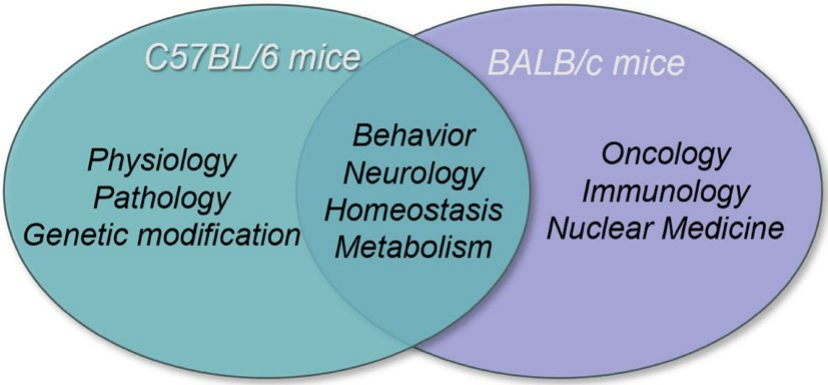
Research and application fields of C57BL/6 and BALB/c mice.

C57BL/6 and BALB/c inbred strains are pivotal in mice research due to their genetic homozygosity and consistent biological response, which make them essential for animal modeling and immunological studies (Figure 1).^1^ However, genetic differentiation—driven by environmental variations, quality control disparities, and genetic drift—can lead to the formation of distinct substrains. These substrains exhibit divergent phenotypes in physiology, biochemistry, and behavioral traits,^2^, ^3^ posing challenges to the interpretation and reproducibility of research findings.

This review compares the origin and differentiation of, as well as genetic and phenotypic variations in, C57BL/6 and BALB/c substrains, discussing advances in their identification and evaluation to guide their precise application in research.

## 2 ORIGIN AND DIFFERENTIATION OF TWO MOUSE STRAINS

**FIGURE 2:**
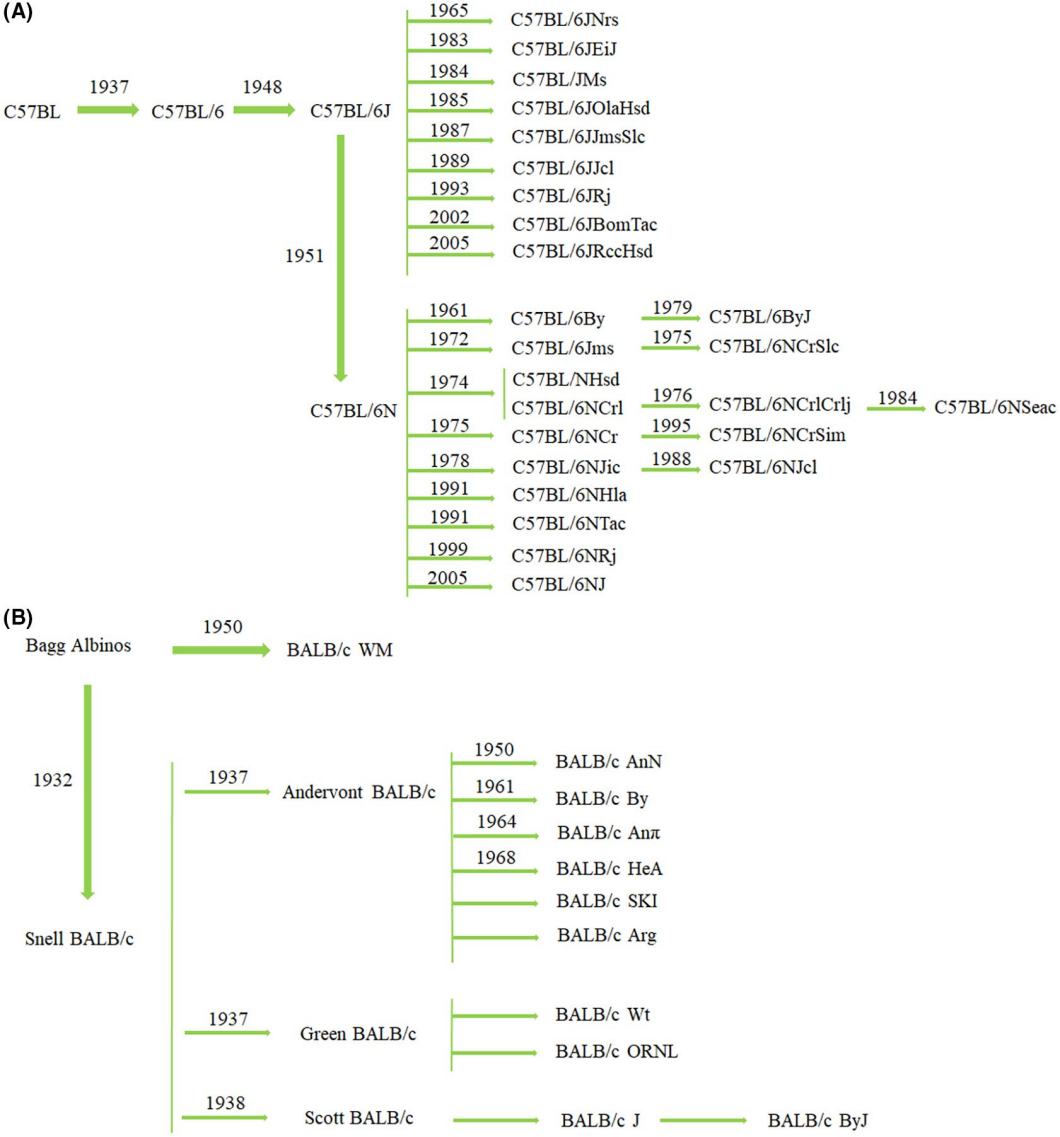
Evolutionary process of the main substrains of C57BL/6 (A) and BALB/c (B).

The C57BL/6 (Figure 2A) and BALB/c (Figure 2B) mice are among the most widely used inbred mouse lines with rich historical backgrounds. Dr. Little established the C57BL strain in 1921. Later, the C57BL/6 mice were brought to Jackson Laboratory (JAX) and subsequently sent to the United States National Institutes of Health (NIH). These mice were raised independently in the two institutes and developed into two major substrains: C57BL/6J (JAX) and C57BL/6N (NIH).^4^, ^5^ With the rising application of mice in basic scientific research, the demand for experimental mice has surged globally. This trend has consequently led to the derivation of more than 20 substrains in C57BL/6 mice worldwide.^6^

In 1913, Bagg obtained the original albino mouse from Ohio businessman and employed a herd breeding method. MacDowell initiated inbreeding of the mice, achieving 26 generations by 1932 and naming the resulting strain as BALB/c. Following the initial establishment, BALB/c mice were transferred to institutions, including JAX, NIH, and Charles River Laboratories (Crl/Cr), at distinct time points, where independent inbreeding led to the development of three substrains: BALB/cJ, BALB/cAnN, and BALB/cCr. In 1935, BALB/cByJ mice were derived from the BALB/cJ through inbreeding for 37 generations of continuous inbreeding, establishing a new BALB/c substrain.^7^

Notably, although phenotypic differences among substrains are well documented, the research community often overlooks the importance of substrain selection.

## 3 ADVANCES IN PHENOTYPIC DIFFERENCES

### 3.1 Behavior and neurophenotype

Most studies investigating phenotypic differences between C57BL/6 substrains have mainly focused on behavioral domains, such as learning and memory, activity and motor function, and anxiety and depression (Table 1).^4^, ^8^ For instance, Amy Ashworth et al.^9^ discovered that C57BL/6J mice exhibited superior adaptability and recognition capabilities in new environments and when presented with novel objects compared to C57BL/6N mice, despite both substrains being sourced from the same supplier. The research team of Oliver Stiedl evaluated the behavioral responses of C57BL/6JOlaHsd and C57BL/6NCrlBR substrains using a contextual and cued fear conditioning paradigm. The experimental results indicate that C57BL/6JOlaHsd mice exhibited higher susceptibility to contextual fear and cued fear compared to C57BL/6NCrlBR mice.^10^ According to Japanese scholar Naoki Matsuo, C57BL/6N mice demonstrated a greater likelihood of receiving pulse stimulation and producing startled behavior compared to C57BL/6J mice.^11^ In social interaction assays, Pinheiro and colleagues discovered that C57BL/6J mice exhibited more active social behaviors compared to C57BL/6N mice, whereas other studies reported stronger motor coordination, specifically in rotation ability, in C57BL/6J mice.^4^, ^11^, ^12^ The C57BL/6 mouse model has been extensively used in alcohol drinking studies. In the alcohol preference experiment conducted by Blum et al., C57BL/6J mice demonstrated higher preference than C57BL/6N. However, in Khisti et al.'s ethanol‐deprivation effect (EDE) study, C57BL/6J mice exhibited a lower EDE compared to C57BL/6NCrl. This phenomenon was further confirmed in a 2007 study by Ramachandra et al., where C57BL/6J mice consumed more alcohol but secreted less dopamine.^13^, ^14^, ^15^ Obesity and metabolic syndrome may influence mechanical pain sensitivity. Mouse models are increasingly being adopted by laboratories to investigate these underlying mechanisms. In the study by Michael A. Cooper et al., compared to C57BL/6J and C57BL/6NIH, C57BL/6CR exhibited the highest body weight, fat mass, and impaired glucose tolerance among the three substrains. Additionally, C57BL/6CR was the only substrain that developed significant mechanical hypersensitivity over an 8‐week period.^16^ Sensitivity to different pain modalities is largely associated with genetic background. In the study by Camron D. Bryant et al., C57BL/6J mice demonstrated enhanced sensitivity to acute thermal nociception compared to C57BL/6N substrains. Through hybridization experiments, the researchers mapped genes associated with pain‐ and inflammation‐related expression traits.^17^

**TABLE 1:** C57BL/6 substrains used for testing various phenotypes.

Descriptions and/or tests	Substrains tested	Results
Adaptability and recognition abilities	C57BL/6J and C57BL/6N	C57BL/6J>C57BL/6N^9^
Susceptibility to situational fear	C57BL/6JOlaHsd and C57BL/6NCrlBR	C57BL/6JOlaHsd>C57BL/6NCrlBR^10^
Likelihood of receiving pulse stimulation	C57BL/6N and C57BL/6J	C57BL/6N>C57BL/6J^11^
Activities of social behaviors	C57BL/6N and C57BL/6J	C57BL/6J>C57BL/6N^4^, ^11^, ^12^
Motor coordination	C57BL/6N and C57BL/6J	C57BL/6J>C57BL/6N^4^, ^11^, ^12^
Alcohol preference	C57BL/6J and C57BL/6N(Crl)	C57BL/6J>C57BL/6N(Crl)^13^, ^14^, ^15^
EDE	C57BL/6J and C57BL/6N(Crl)	C57BL/6N(Crl)>C57BL/6J^14^, ^15^
High‐fat diet‐induced mechanical sensitivity	C57BL/6J, C57BL/6NIH, and C57BL/6CR	C57BL/6CR>C57BL/6NIH/C57BL/6J^16^
Sensitivity to thermal nociception	C57BL/6N and C57BL/6J	C57BL/6J>C57BL/6N^17^

Abbreviation: EDE, ethanol‐deprivation effect.

BALB/c mice are commonly used to investigate neuropsychiatric phenotypes (Table 2). In studies of aggressive behavior among BALB/c substrains, BALB/cJ exhibited greater aggression than BALB/cByJ.^18^, ^19^ However, Laura J. Sittig attained conflicting results regarding the aggression of these two substrains of mice during her resident invader test on BALB/cByJ and BALB/cJ. This disparity may be attributed to variations in study parameters or research conditions.^20^ Amanda Jager's team conducted a study on the influence of negative feedback and reward sensitivity on cognitive flexibility. Their findings showed that BALB/cJ mice exhibited less sensitivity to negative feedback and greater cognitive flexibility under reward conditions.^21^ These characteristics in BALB/cJ mice make them a valuable model for further study of the molecular and neural basis of conduct disorder in patients. Furthermore, Jacob A. Beierle's research on thermal sensitivity and mechanical stimulation revealed that BALB/cByJ mice demonstrated increased sensitivity to a 53.5°C hot plate and to mechanical stimulation compared to BALB/cJ mice.^22^

**TABLE 2:** BALB/c substrains used for testing various phenotypes.

Descriptions and/or tests	Substrains tested	Results
Aggression	BALB/cJ and BALB/cByJ	BALB/cJ>BALB/cByJ^18^, ^19^ BALB/cByJ>BALB/cJ^20^
Influence of negative feedback	BALB/cJ and BALB/cByJ	BALB/cByJ>BALB/cJ^21^
Reward sensitivity	BALB/cJ and BALB/cByJ	BALB/cJ>BALB/cByJ^21^
Sensitivity of thermal and mechanical stimulation	BALB/cJ and BALB/cByJ	BALB/cByJ>BALB/cJ^22^

### 3.2 Homeostasis and metabolic phenotypes

Due to the lack of the niacinamide mononucleotide transferase (*Nnt*) gene exon for niacinamide mononucleotide transferase, C57BL/6J mice exhibited reduced glucose tolerance compared to other C57BL/6 substrains (Table 3). As a result, they have been frequently used as a model for metabolic diseases and diet‐induced obesity.^23^ In vivo studies have demonstrated that C57BL/6J and C57BL/6N substrains exhibited varying insulin responses under conditions of high‐fat feeding, whereas no difference was observed in insulin response after low‐fat diet.^24^, ^25^ There was variability in insulin response to high‐fat feeding in C57BL/6N, but it was absent in C57BL/6J.^24^ In addition, high‐diet intervention led to an increase in baseline and high‐fat diet‐induced vascular superoxide in C57BL/6J mice compared to C57BL/6N mice. Furthermore, C57BL/6J mice displayed significantly higher levels of atherosclerotic plaque formation.^26^ A study on the effects of an oat bran diet found that C57BL/6NCrl mice had a higher likelihood of experiencing sustained reductions in plasma cholesterol concentration and lipoprotein levels compared to C57BL/6JBomTac mice.^27^ Additionally, the distinct mitochondrial functional characteristics and pro‐oxidative mitochondrial phenotype of different C57BL/6 substrains were linked to the differential expression of *Nnt* gene, thereby affecting the redox balance of the mice.^28^ In a free feeding state, the C57BL/6J group exhibited reduced gas exchange and lower energy expenditure compared to the C57BL/6N group.^29^ In addition to disparities in oxidation and glucose metabolism, clinical chemistry, hematology, and iron‐related parameters differed between the two substrains.^23^, ^30^

**TABLE 3:** Potential applications of C57BL/6 and BALB/c substrains.

Strains	Substrains	Descriptions	Potential applications
C57BL/6	/	C57BL/6 is more susceptible to HTN due to differential expression of *Nnt* ^28^	As a model to study HTN
	C57BL/6J	C57BL/6J exhibits reduced glucose tolerance^23^	As a model for metabolic diseases and diet‐induced obesity
		C57BL6/J exhibits higher levels of atherosclerotic plaque formation on a high‐fat diet^26^	As a model for high diet inducing atherosclerotic
		C57BL/6J exhibits lower levels of basal metabolism^29^	As a model for studying the influencing factors of basal metabolism
	C57BL/6NCrl	C57BL/6NCrl is more prone to decrease plasma cholesterol concentration and lipoprotein levels in oat bran diet^27^	As a model for studying the mechanism of oat bran diet in reducing cholesterol and lipoprotein levels
	C57BL/6NJ	C57BL/6NJ shows no increase in insulin release after high‐fat feeding^24^	As a model for high‐fat diet‐induced diabetes
BALB/c	BALB/cJ	BALB/cJ exhibits lower resistance to atherosclerosis in high‐fat diet^32^	As a model for high‐fat diet‐inducing atherosclerotic plaque

Abbreviations: HTN, hypertension; *Nnt*, niacinamide mononucleotide transferase.

In their metabolic study on BALB/c mice, Perincheri et al.^31^ discovered a significant reduction in the expression of zinc finger and homeobox 2 (*Zhx2*) genes, which encode transcription factors in most BALB/c substrains. They theorized that the phenotypic differences influenced the decline in major urinary protein (Mup) levels, which act as regulators of glucose and lipid metabolism, resulting in lower serum lipid levels and increased resistance to atherosclerosis in high‐fat diets.^32^

### 3.3 Immunity and disease susceptibility phenotypes

Compared to other research domains, the investigation of immune system phenotype in mouse substrains has a relatively short history (Table 4). In 2007, Oleg Garifulin reported that C57BL/6ByJ, which carries a deficiency in the interferon‐β (IFN‐β) signaling pathway, exhibited significantly enhanced resistance to *Listeria monocytogenes* infection compared to other C57BL/6 substrains.^33^ Subsequent studies have revealed heterogeneity between C57BL/6J and C57BL/6N substrains in three key aspects: the response of neutrophilic recruitment to inflammatory stimuli, the expression level of non‐exogenous endogenous retrovirus (ERV), and susceptibility to influenza A virus between C57BL/6J and C57BL/6N substrains.^34^, ^35^, ^36^

**TABLE 4:** Immunity and disease susceptibility phenotypes and mechanisms.

Substrains	Phenotypes	Mechanisms
C57BL/6ByJ	More resistant to *Listeria* monocytogenes infection	Irf3 mRNA was inefficiently spliced in C57BL/6ByJ^33^
C57BL/6J	Increased susceptibility to bacterial infection	NLRP12‐deficient macrophages have impaired CXCL1 production^34^
	More susceptible to inflammation‐associated disease caused by influenza A virus	Six genes exhibit increased transcript levels in influenza virus‐infected mouse lungs^35^
BALB/cJ BALB/cAnNCr BALB/cByJ BALB/cCum	BALB/cJ and BALB/cAnNCr mice are susceptible, whereas BALB/cByJ and BALB/cCum are resistant to Theiler's murine encephalomyelitis virus (TMEV)‐induced demyelinating disease	Differentiation of several distinct loci, including H‐2D on chromosome 17, *Tmevd‐1* on chromosome 6, and *Tmevd‐2* on chromosome 3, and more than one non‐H‐2 loci^37^
BALB/cByJ	BALB/cByJ mice are more susceptible to *Plasmodium yoelii* preerythrocytic infection than BALB/cJ mice	Hepatocyte‐specific differences in mRNA abundance for numerous genes^38^
BALB/c	Arthritis‐prone	A number of the known genes (and SNPs) are associated with immune responses and/or arthritis^39^
	Various commercial suppliers had diverse antibody profiles concerning seasonal trivalent influenza vaccine and adjuvant immunity	Genetic drift^41^
BALB/cJ	Graves' hyperthyroidism‐prone	The importance of other genes, such as Qa‐2 region of class Ib molecule in addition to MHC class II, in the susceptibility of Graves' hyperthyroidism^40^

Abbreviations: MHC, major histocompatibility complex; mRNA, messenger RNA; SNPs, single‐nucleotide polymorphisms.

The BALB/c mouse strain is widely used in immunological research, with varying immune responses observed among different substrains. Nicholson investigated the susceptibility of four BALB/c substrains to induced demyelinating disease caused by Theiler's murine encephalomyelitis virus (TMEV), immune‐mediated inflammatory demyelinating disease, and human multiple sclerosis experimental models.^37^ His findings revealed that BALB/cJ and BALB/cAnNCr mice were susceptible to these diseases. Although BALB/cByJ and BALB/cCum were initially resistant, BALB/cByJ mice exhibited greater susceptibility to *Plasmodium yoelii* infection during early hepatocyte infection compared to BALB/cJ mice.^38^ In a study on inducing arthritis and spondylitis using an optimal dose of cartilage proteoglycans, Farkas B. reported substrain‐dependent differences in disease visibility and a significant number of differentially expressed genes in the 11 BALB/c mouse substrains.^39^ Studies indicate that BALB/cBy mice are more sensitive to hyperthyroidism induction compared to BALB/c mice due to their significantly higher antibody‐blocking activity.^40^ Genetic drift can occur during the maintenance breeding of mice due to various breeding environments and feeding methods used by different breeders, resulting in the development of distinct substrains. Poyntz et al. discovered that BALB/c substrain mice from various commercial suppliers had diverse antibody profiles concerning seasonal trivalent influenza vaccine and adjuvant immunity. In instances of BALB/c substrain with low antibody response, there was severe disruption of the class transition (C‐S) within the T‐helper cells and germinal center B cells, leading to fewer germinal center responses resulting in poor formation of antigen‐specific immunoglobulin G (IgG) plasma cells and fewer IgG antibody types.^41^ Therefore, to ensure comparable experimental results in the evaluation of vaccine immunogenicity and protection using mice, it is advisable to use mice from the same supplier for the experiments to avoid heterogeneity‐induced noncomparability.

## 4 ADVANCES IN GENETIC DIFFERENCES

Each generation of mice undergoes over 100 spontaneous mutations, with a subset becoming heritable. As mutations accumulate, fixed mutations are maintained. When fixed mutations increase to a certain level, a new mouse substrain is formed.^42^ These genotypic differences between substrains can modulate the process of protein translation and expression, thereby influencing mouse biological characteristics and observable phenotypes of mice.

Currently, many studies have reported genetic differences between C57BL/6 and BALB/c substrains. Decades of genetic drift have driven quantifiable genetic differences, including a mutation in the *Nnt* gene that reduces insulin secretion in C57BL/6J, which has a wild‐type allele and is unaffected.^43^ Whole‐genome sequencing (WGS) and RNA sequencing have also demonstrated additional genetic variation between mouse substrains. Between C57BL6/N and C57BL/6J mice, the Simon team identified 34 coding single‐nucleotide polymorphisms (SNPs), two coding small insertion losses, 146 noncoding SNPs, 54 noncoding small insertion deletions, and 43 structural variants (including *Nnt* mutations). Mortazavi et al. also found varying levels of SNPS, short tandem repeats, and structural variation among nine C57BL/6 substrains from four suppliers.^44^

Studies on different BALB/c substrains have shown that although BALB/cJ and BALB/cByJ mice have the same MHC haplotype H‐2d, there is a difference in the Qa‐2 region of Ib molecules. The Qa‐2 region of Ib molecules was intact in BALB/c mice (Qa‐2+), whereas this region was missing in BALB/cBy mice (Qa‐2‐).^10^, ^45^ In addition, Japanese researcher Ikuo Miura discovered six SNPs that could be used to identify four different substrains of BALB/c mice: BALB/cJ, BALB/cAJcl, BALB/cAnNCrlCrlj, and BALB/cCrSlc.^46^

## 5 DISCUSSION

Research on mouse substrains is more advanced in Europe and America, attributed to longer breeding histories and continuous innovation in genetic quality control. The techniques for ensuring genetic quality in mice have evolved from morphological markers, chromosome analysis, protein‐based assay, and immunological methods to modern molecular biology techniques. Festing first identified the genetic background of the mice using mandibular measurements.^47^ Roderick et al. proposed the use of isozyme alleles for genetic monitoring in animals^48^ followed by Whitmore et al., who introduced skin transplantation for genotype identification after the discovery of the major histocompatibility complex (MHC).^49^ With the advancement of molecular biology technology, techniques such as DNA fingerprinting, genetic mapping, and microsatellite repeat sequence detection have been applied to mouse genetic quality control. In recent years, SNP marker technology has emerged as the primary method for evaluating genetic quality in mice, leveraging its advantages of rapidity, high throughput, and direct gene variation detection. Internationally renowned laboratory animal suppliers, such as JAX, Crl, Taconic, and Harlan, employ these molecular biogenetic markers for breeding and quality control of mouse substrains.^50^ Although protein‐level biochemical marker detection remains the national standard for mouse strain quality control in China, its low detection efficiency and accuracy have prompted research teams, such as Professor Chen Zhenwen's research team and the Shanghai Laboratory Animal Center, to adopt SNP technology for characterizing domestic mouse resources.^51^, ^52^, ^53^ Notably, microsatellite biochemical markers are still recommended for genetic quality assessment of experimental animals in specific scenarios.^54^

Enhancing genetic testing techniques and standards is imperative for the precise utilization of mouse substrains. Advanced substrain evaluation technology facilitates the establishment of efficient breeding system, enabling the elucidation of distinct biological and genetic characteristics across different substrains of mice. This meticulous screening process, in turn, identifies optimized mouse substrains tailored to specific research applications. Implementing a substrain optimization strategy is crucial to ensure the stability, reliability, and reproducibility of scientific investigations. By minimizing phenotypic and genotypic variations arising from substrain divergence, this approach mitigates research inconsistencies and enhances experimental rigor, thereby fostering more robust and translatable findings. Proper execution of this framework is fundamental to safeguarding the validity and replicability of research outcomes.^55^

## AUTHOR CONTRIBUTIONS

**Lan Zhao:** Writing – original draft; writing – review and editing. **Jie Wei:** Funding acquisition; writing – review and editing. **Bingfei Yue:** Conceptualization; funding acquisition; writing – review and editing.

## FUNDING INFORMATION

This study was funded by the National Key R&D Program of China (grant number: 2021YFF0703200) and the Key Technology Fund of the National Institutes for Food and Drug Control (GJJS‐2022‐1‐5).

## CONFLICT OF INTEREST STATEMENT

The authors declared no competing financial or commercial interests.

## ETHICS STATEMENT

None.
